# Characterization of genes associated with *TGA7* during the floral transition

**DOI:** 10.1186/s12870-021-03144-w

**Published:** 2021-08-11

**Authors:** Xiaorui Xu, Jingya Xu, Chen Yuan, Yikai Hu, Qinggang Liu, Qianqian Chen, Pengcheng Zhang, Nongnong Shi, Cheng Qin

**Affiliations:** Research Centre for Plant RNA Signaling, College of Life and Environmental Sciences, Hangzhou Normal University, 311121 Hangzhou, China

**Keywords:** Flowering time, TGA7, Delayed flowering, *Arabidopsis*, Transcriptome

## Abstract

**Background:**

The TGACG-binding (TGA) family has 10 members that play vital roles in *Arabidopsis thaliana* defense responses and development. However, their involvement in controlling flowering time remains largely unknown and requires further investigation.

**Results:**

To study the role of *TGA7* during floral transition, we first investigated the *tga7* mutant, which displayed a delayed-flowering phenotype under both long-day and short-day conditions. We then performed a flowering genetic pathway analysis and found that both autonomous and thermosensory pathways may affect *TGA7* expression. Furthermore, to reveal the differential gene expression profiles between wild-type (WT) and *tga7*, cDNA libraries were generated for WT and *tga7* mutant seedlings at 9 days after germination. For each library, deep-sequencing produced approximately 6.67 Gb of high-quality sequences, with the majority (84.55 %) of mRNAs being between 500 and 3,000 nt. In total, 325 differentially expressed genes were identified between WT and *tga7* mutant seedlings. Among them, four genes were associated with flowering time control. The differential expression of these four flowering-related genes was further validated by qRT-PCR.

**Conclusions:**

Among these four differentially expressed genes associated with flowering time control, *FLC* and *MAF5* may be mainly responsible for the delayed-flowering phenotype in *tga7*, as *TGA7* expression was regulated by autonomous pathway genes. These results provide a framework for further studying the role of *TGA7* in promoting flowering.

**Supplementary Information:**

The online version contains supplementary material available at 10.1186/s12870-021-03144-w.

## Background

TGACG-binding (TGA) transcription factors (TFs) belong to the bZIP TF family. There are 10 members in the TGA family, and they play essential roles in *Arabidopsis thaliana* defense responses and development [1–3]. These TGAs can interact with non-repressor of pathogenesis-related gene 1 (*NPR1*), which is involved in salicylic acid (SA)-mediated gene expression (similar to *PR-1*) and disease resistance [4, 5]. These TGAs bind to *cis*-regulatory TGACG elements [6], and this element is present in *PR1* promoters, which are required for *PR1* gene expression in response to SA and interact with NPR1 [4, 7–9]. However, NPR1 cannot bind directly to the *PR-1* promoter, but is recruited to the promoter by its physical interaction with TGAs to regulate the expression of *PR-1* [4, 6–9].

The NPR1 protein interacts with 7 of the 10 *Arabidopsis* TGAs [7, 8, 10]. These seven TGAs are further classified into three subclades, with clade I containing TGA1 and TGA4; clade 2 containing TGA2, TGA5, and TGA6; and clade III containing TGA3 and TGA7 [11]. In *Arabidopsis*, only TGA1 and TGA4 interact with NPR1 in SA-induced leaves, whereas the other TGAs constitutively interact with NPR1 [12]. Thus, all seven TGAs are the important components of the plant defense system.

In addition to their involvement in plant defenses, TGAs also act in plant development. For instance, when grown under low-nitrate conditions, *tga1/tga4* shows an altered root architecture [13, 14]. *TGA1* and *TGA4* are also expressed around flower organ boundaries and are required for inflorescence architecture, meristem maintenance, and flowering [3].

In this study, we showed that TGA7 plays an important role in flowering time control. The loss of *TGA7* function delayed flowering in *Arabidopsis*. To reveal the molecular mechanisms of *TGA7* in flowering time control, the transcriptomic changes between WT and *tga7* mutant seedlings at 9 days after germination (DAG) were analyzed by RNA-sEq. A total of 325 differentially expressed genes (DEGs) were identified, and 4 DEGs were associated with flowering time pathways. These results provide insights into the genes potentially related to flowering time control in the *tga7* mutant and will be useful for further studies on the molecular mechanisms of *TGA7* in floral transition.

## Methods

### Plant materials

*Arabidopsis* plants were grown in soil under long-day (LD; 16-h/8-h, light/dark) or short-day (SD; 8-h/16-h, light/dark) conditions at 23 °C. Mutants *gi-1*, *co-9*, *ft-10*, *svp-41*, Col:*FRI*^*SF2*^ (*FRI-Col*), *fld-3*, and *fve-4* were all in the Columbia background [15, 16]. *fpa-7* (SALK_138449), *fca-2* (SALK_057540), *flk-1* (SALK_007750), and *tga7* (CS89835) seeds were bought from the Arabidopsis Biological Resource Center (http://www.arabidopsis.org/).

### Cleaved Amplified Polymorphic Sequences (CAPS) analysis

A 689-bp DNA fragment of the *tga7* mutant or wild-type (WT) was amplified using the following primers: Forward, 5′-TAAAGTTATCGCAGTTAGAGC-3′ and Reverse, 5′-CCGCATCAATCACAATG-3′. PCR was carried out for 40 cycles of 95 °C for 30 s, 58 °C for 30 s, and 72 °C for 1 min. Then, the PCR products were digested by *Eco*RV and separated on 1 % agarose-TAE gels.

### Plasmid construction and transgenic plant generation

To construct *35 S:TGA7*, the *TGA7* coding sequence was amplified and then cloned into the binary vector *pCAMBIA1300-35 S*. The primers used for plasmid construction are listed in Additional file 1. Transgenic plants were generated through *Agrobacterium tumefaciens*-mediated transformation using the floral-dipping method. Transformants containing *35 S:TGA7* were selected on MS medium supplemented with hygromycin (30 mg L^− 1^).

### Total RNA isolation

The isolation of total RNA was performed using an RNAprep Pure Plant Kit (TIANGEN, Beijing, China) in accordance with the manufacturer’s instructions. DNase I was added to the mixture to eliminate genomic and plastid DNA.

### mRNA library construction

Total RNA was analyzed using a NanoDrop and Agilent 2100 bioanalyzer (Thermo Fisher Scientific, MA, USA). To purify mRNA, oligo(dT) magnetic beads were used. Then, the mRNA was sheared into small fragments in the fragmentation buffer. The first-strand cDNA was synthesized by reverse transcription using random hexamer primers, and the second-strand cDNA was synthesized by DNA polymerase. Afterwards, adapters were added to the double-stranded cDNA. To amplify the cDNA fragments, PCR was performed, and the resulting PCR products were purified and dissolved in elution buffer. Then, the PCR products were heat-denatured to produce the final library. The sequencing was performed on a BGIseq500 platform (BGI-Shenzhen, China). The transcriptome data sets have been submitted to the NCBI (accession number PRJNA649868).

### De novo assembly and functional annotation of sequencing

The transcriptome data were filtered and analyzed in accordance with a previously published protocol with minor modifications [17]. A differential expression analysis was performed, and the significance levels of gene ontology (GO) terms were all determined, using *Q* value ≤ 0.05.

### qRT-PCR

For the expression analysis, 1 µg RNA was used for reverse transcription. The cDNA was synthesized using a FastKing gDNA Dispelling RT SuperMix kit (TIANGEN) in accordance with the manufacturer’s instructions. qRT-PCR was performed using the UltraSYBR Mixture (with ROX; CWBio, Beijing, China) and the CFX96 real-time PCR detection system (Bio-Rad). The expression levels of detected genes were normalized to *TUB2* expression. Error bars denote standard deviations of three biological replicates [18]. The primers used for the expression analysis are listed in Additional file 1.

## Results

### Regulation of *Arabidopsis* flowering time by *TGA7*

**Fig. 1:**
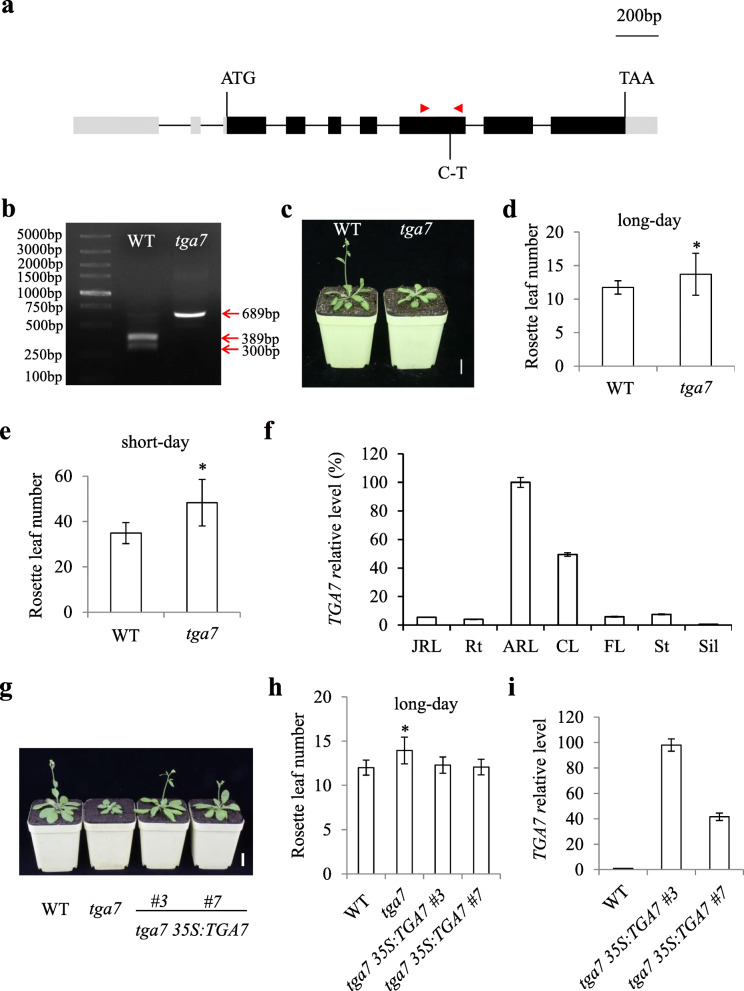
*TGA7* regulates flowering time in *Arabidopsis*. **a** The structure of the TGA7 coding region. Black boxes, gray boxes, and black lines represent exons, untranslated regions, and introns, respectively. The point mutation is shown underneath. Red arrowheads indicate the positions of primers used in b. **b** The cropped gels of the CAPS analysis of the wild-type and *tga7* mutant. Genomic DNAs of the wild-type and *tga7* mutant were amplified using the CAPS markers list in Additional file 1, and then, the PCR products were digested with *Eco*RV. **c***tga7* showed a delayed-flowering phenotype under LD conditions. Scale bar = 2 cm. **d** and **e** Flowering times of *tga7* grown under LD (**d**) and SD (**e**) conditions. Values are from at least 10 plants showing specific genotypes. Asterisks indicate significant differences in flowering time between the WT and *tga7* mutant (Student’s *t* test, *p* ≤ 0.05). **f** The expression level of *TGA7* in various tissues of WT plants was analyzed by qRT-PCR (*n* = 3, ±standard deviations). JRL, juvenile rosette leaves; Rt, roots; ARL, adult rosette leaves; CL, cauline leaves; FL, flowers; St, inflorescence stems; Sil, siliques. **g***tga7 35 S:TGA7* exhibited a flowering time comparable to that of WT plants under LD conditions. Scale bar = 2 cm. **h** Flowering time of *tga7 35 S:TGA7* grown under LD conditions. Values are representative of at least 15 plants showing specific genotypes. Asterisks indicate significant differences between the WT and *tga7* mutant in flowering time (Student’s *t* test, *p* ≤ 0.05). **i** The *TGA7* expression levels in independent *TGA7-*overexpression lines at 9 DAG. Error bars denote standard deviations

To reveal the function of *TGA7* in controlling flowering time, we analyzed the *TGA7* phenotype using a *tga7* mutant that contained a point mutation in the seventh exon (Fig. 1a). The C to T mutation led to the loss of an *Eco*RV site in the *TGA7* gene and resulted in an amino acid change from Ser to Leu in the TGA7 protein (Fig. 1a, b, Additional file 2). All the *tga7* mutant plants had delayed flowering compared with wild-type (WT) seedlings under both LD and SD conditions (Fig. 1c–e), suggesting that *TGA7* promotes flowering independently of the day length conditions. To determine whether the point mutation in the *TGA7* gene is truly responsible for the observed phenotype, the mutants were backcrossed with the WT (Col-0). The F_1_ seedlings showed a WT phenotype (Additional file 3a, b), and F_2_ seedlings showed a segregation ratio of 3:1 (31:12, WT:*tga7* phenotypes, χ^2^ = 0.19 < χ^2^_0.05_ = 3.84; *P* > 0.05). We then analyzed all the 12 F_2_ seedlings having the *tga7* mutant phenotype. These 12 F_2_ seedlings all displayed a homozygous point mutation in the *TGA7* gene (Additional file 3c, d). We then transformed the *tga7* mutant with a construct containing the coding sequence of *TGA7* driven by the 35 S promoter. Two independent *tga7 35 S:TGA7* transgenic lines exhibited flowering times comparable to those of WT plants (Fig. 1 g–i), indicating that TGA7 was responsible for the flowering phenotype of the *tga7* mutant and that excess amounts of *TGA7* do not further accelerate flowering.

We then examined *TGA7* expression in different tissues of WT plants by qRT-PCR and found that the highest *TGA7* expression occurred in adult rosette leaves, whereas there was almost no expression of *TGA7* in siliques (Fig. 1f).

### Autonomous and thermosensory pathways regulate *TGA7* expression

**Fig. 2:**
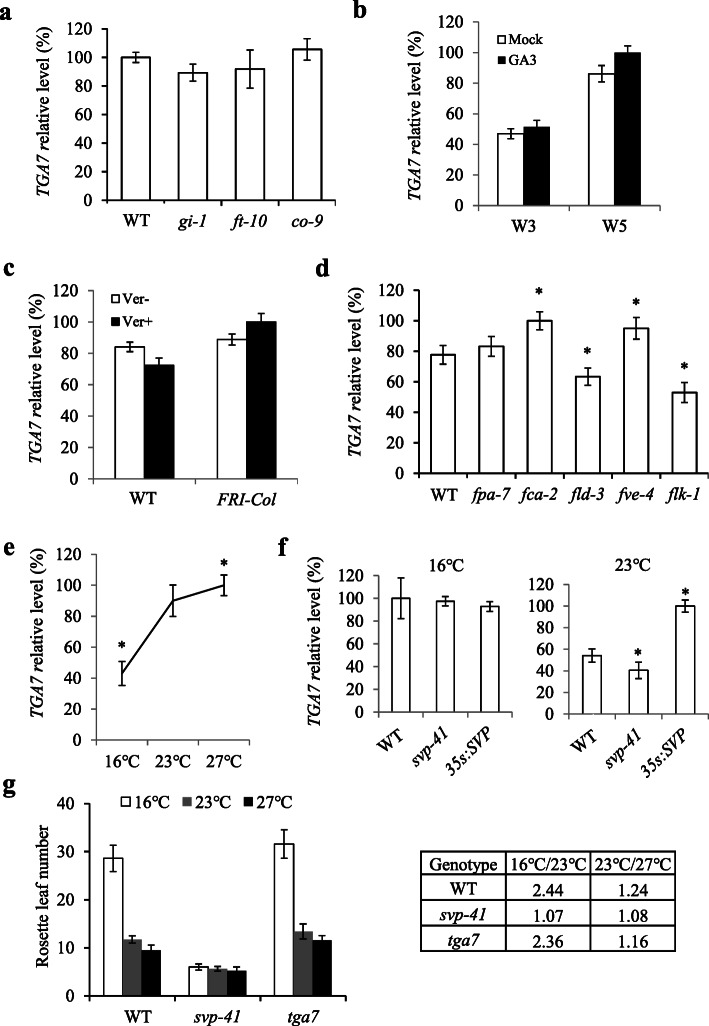
*TGA7* expression is regulated by several pathways. **a ***TGA7* expression in photoperiod-pathway mutants at 9 DAG. **b ***TGA7* expression after a gibberellin (GA) treatment. WT seedlings were grown under SD conditions for 2 weeks and then treated with 100 µM GA_3_ or 0.1 % ethanol weekly. Seedlings treated for 3 (W3) and 5 weeks (W5) were collected for further analyses. **c ***TGA7* expression after a vernalization treatment. The seedlings were vernalized at 4 °C for 8 weeks. The 9-day-old seedlings were collected for further analyses. **d ***TGA7* expression in autonomous-pathway mutants at 9 DAG. **e** The *TGA7* expression level in WT seedlings grown at 16℃, 23℃, and 27℃ under LD conditions until 9 DAG. **f ***TGA7* expression in WT, *svp-41*, and *35 S:SVP* plants grown at 16 and 23 °C under LD conditions until 9 DAG. Asterisks indicate significant differences (Student’s *t* test, *p* ≤ 0.05). **g** Flowering times of wild-type, *svp-41*, and *tga7* plants grown at 16 °C, 23 °C, and 27 °C under LD conditions. The ratios of flowering time between 16 and 23 °C (16 °C/23°C) and between 23 and 27 °C (23 °C/27°C) for all the genotypes are indicated in the attached table. Values were scored from at least 15 plants of each genotype. Error bars indicate standard deviations

Because TGA7 is involved in floral transition, we examined which flowering genetic pathways may be involved in flowering time control. The expression of *TGA7* remained steady in the photoperiod pathway mutants (Fig. 2a), and the phenotype of the *tga7* mutant was delayed flowering under LD and SD conditions (Fig. 1c–e), suggesting that *TGA7* may not be involved in the photoperiod pathway. In addition, there were almost no effects on *TGA7* expression after a gibberellin treatment (Fig. 2b). In both WT and *FRI-Col* plants, a vernalization treatment did not alter *TGA7* expression (Fig. 2c). These observations suggest that the gibberellin and vernalization pathways did not influence *TGA7*. By contrast, in the autonomous pathway mutants *fca-2* and *fve-4*, the *TGA7* expression level increased, whereas it decreased in *fld-3*and *flk-1* (Fig. 2d), suggesting that the autonomous pathway may affect *TGA7* expression.

The *SVP* gene plays crucial roles in the thermosensory pathway, and the *svp-41* mutant displays a steady flowering phenotype under different temperature conditions [19]. We also analyzed *TGA7* expression at different temperatures. The *TGA7* expression increased along with the temperature (Fig. 2e). Furthermore, *TGA7* expression was steady in WT, *svp-41*, and *35 S:SVP* plants at 16℃, whereas *TGA7* expression was higher in *35 S:SVP* but lower in *svp-41* at 23℃ (Fig. 2f). These findings demonstrate that the thermosensory pathway may also regulate *TGA7* expression at ambient temperatures. In addition, *tga7* flowered in a temperature-sensitive manner from 16 to 23 °C, but also flowered in a partial temperature-insensitive pattern from 23℃ to 27℃ (Fig. 2g). Thus, TGA7 may partially mediate the effect of the thermosensory pathway on flowering time.

### Transcriptomes of WT and *tga7* mutant seedlings

To understand how *TGA7* affects flowering time, we identified genes downstream of *TGA7* that might be involved in its role in promoting flowering. The RNA-seq analyses of WT and *tga7* mutant seedlings were performed, and mRNAs were extracted, with three biological replicates, from WT and *tga7* mutant seedlings at 9 DAG. In total, six RNA-seq libraries were constructed for transcriptome sequencing.

**Fig. 3:**
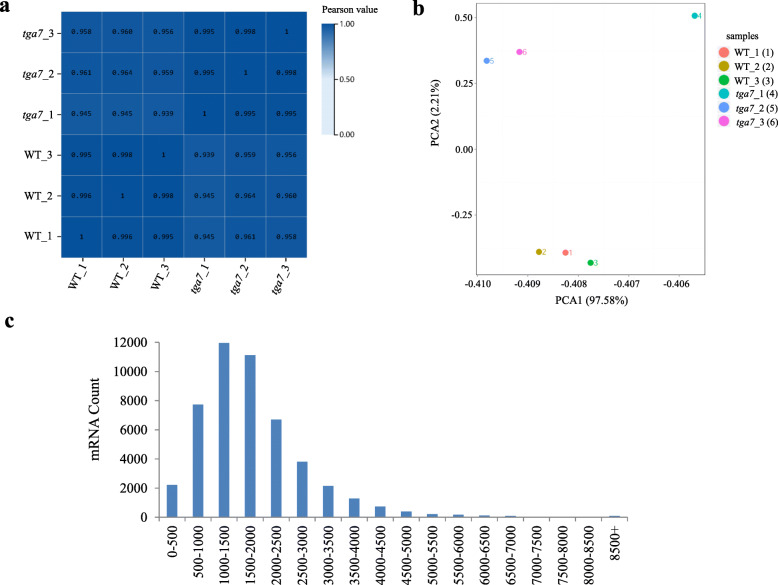
Transcriptomes of WT and *tga7* mutant seedlings. **a** Pair-wise Pearson’s correlation coefficients analysis showing that the sequencing data from three replicates of two samples are highly repeatable. **b** Principal components analysis of the transcriptomes. **c** The size distributions of mRNAs of the transcriptomes

The raw data were qualified and filtered, yielding approximately 6.67 Gb of sequence data per library (Additional file 4). A Pair-wise Pearson’s correlation coefficients analysis of three replicates of each sample indicated that the sequencing data were highly repeatable (Fig. 3a). To gain an overview of the variations among the sequencing data, a principal components analysis (PCA) was performed, and the values of PC1 and PC2 were 97.58 and 2.21 %, respectively (Fig. 3b). The PCA clearly separated the six RNA-seq libraries into two groups, WT and *tga7* mutant. The size distributions of the mRNAs are shown in Fig. 3c. The majority of mRNAs (84.55 %) were between 500 bp and 3,000 bp, and only 1.60 % of the mRNAs were > 5,000 bp.

### Identification of DEGs between WT and *tga7* mutant seedlings

**Fig. 4:**
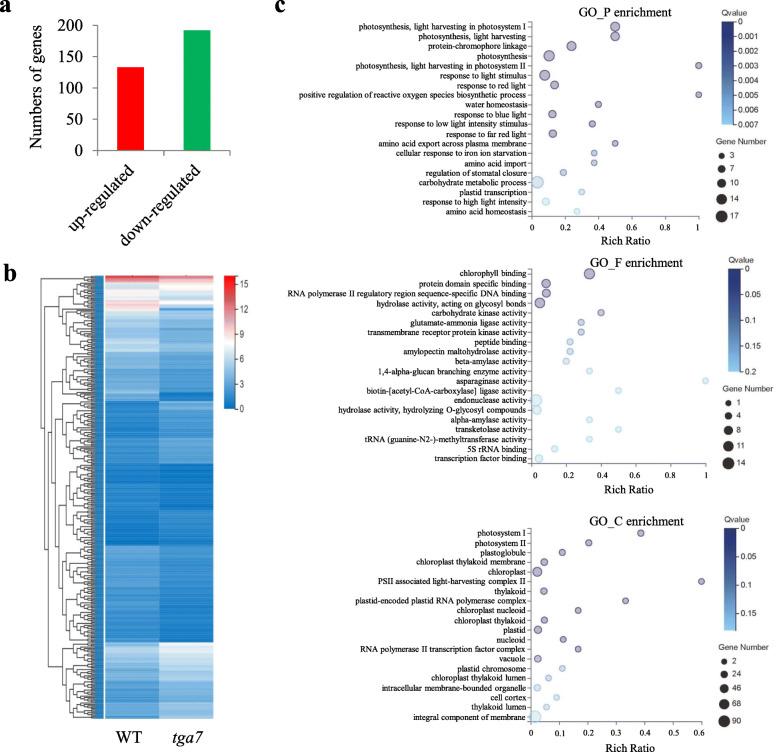
Transcriptional profiles of WT and *tga7* mutant seedlings at 9 DAG. **a** The numbers of genes that were up-regulated or down-regulated between WT and *tga7* mutant seedlings at 9 DAG. **b** Expression profiles of the DEGs between WT and *tga7* mutant seedlings at 9 DAG shown using a heatmap. **c** GO term enrichment analysis of the DEGs between WT and *tga7* mutant seedlings

The reads per kb per million reads values were calculated to determine the DEGs between WT and *tga7* mutant seedlings at 9 DAG. In total, 325 DEGs were identified, of which 133 genes were induced and 192 genes were repressed (Fig. ​4a). Among the 325 DEGs, *AT3G55970*, *AT5G45570*, *AT5G44590*, *AT5G44440*, and *AT4G12480* were the most up-regulated genes, whereas *AT3G01345*, *AT4G36700*, *AT3G56980*, *AT5G28520*, and *AT4G36700* were the most down-regulated genes. The heatmap in Fig. 4b shows the expression profiles of the DEGs between WT and *tga7* mutant seedlings. A GO term enrichment analysis of these DEGs was performed and the top five most represented GO terms in biological process were “photosynthesis, light harvesting in photosystem I”, “photosynthesis, light harvesting”, “protein-chromophore linkage”, “photosynthesis”, and “photosynthesis, light harvesting in photosystem II”. In molecular function, they were “chlorophyll binding”, “protein domain specific binding”, “RNA polymerase II regulatory region sequence-specific DNA binding”, “hydrolase activity, acting on glycosyl bonds”, and “carbohydrate kinase activity”. In cellular component, the top five most represented GO terms were “photosystem I”, “photosystem II”, “plastoglobule”, “chloroplast thylakoid membrane”, and “chloroplast” (Fig. 4c).

### Identification of key flowering time-related DEGs

A large number of genes are flowering time-related and play vital roles in the floral transition, an important turning point from vegetative to reproductive growth [20–22]. Among the 325 DEGs identified between WT and *tga7* mutant seedlings (Figs. 4), 4 DEGs were involved in flowering time pathways. The expression levels of *FLC*, *MAF5*, and *SMZ* were up-regulated, whereas that of *NF-YC2* was down-regulated in *tga7* mutant seedlings, compared with WT seedlings (Additional file 5).

### Validation of the expression levels of flowering time-related DEGs

**Fig. 5:**
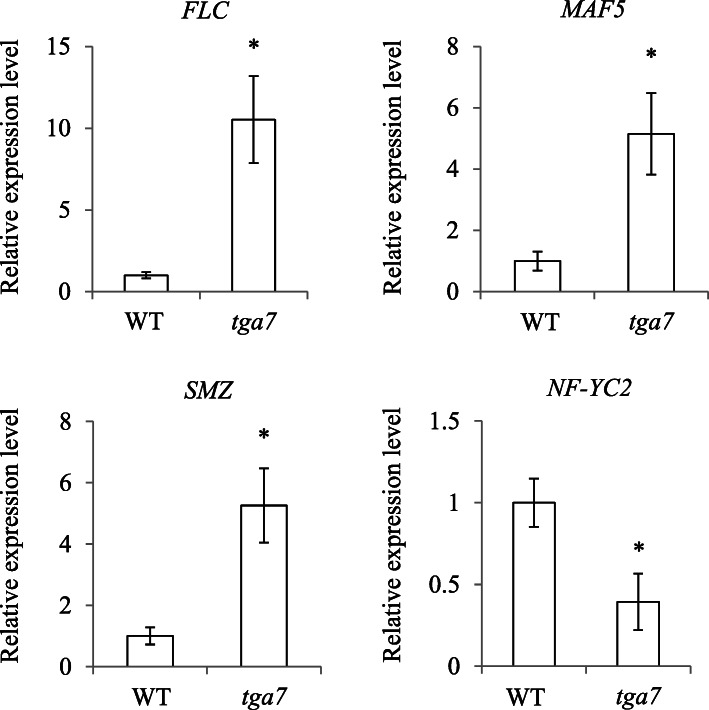
Quantitative real-time PCR validation of flowering time-related DEGs. Expression levels in all the panels were determined by qRT-PCR and then normalized to *TUB2* expression. The data are from three independent replicates. Error bars denote significant differences. Asterisks indicate significant differences among samples (Student’s *t* test, *p* ≤ 0.05)

To validate the expression of the four flowering time-related DEGs (*FLC*, *MAF5*, *SMZ*, and *NF-YC2*) identified by RNA-seq (Additional file 5), three independent biological duplicates of WT and *tga7* mutant seedlings collected at 9 DAG were analyzed by qRT-PCR. The expression levels and trends of the four flowering-related DEGs were consistent with the RNA-seq results (Fig. 5), which indicated that the RNA-seq data are reliable.

## Discussion

In the present study, *Arabidopsis* that had lost *TGA7* function showed a delayed-flowering phenotype (Fig. 1). To uncover the role of *TGA7* in flowering time control, transcriptomic analyses between WT and *tga7* mutant seedlings at the same developmental stage (9 DAG) revealed 325 DEGs, among which *NF-YC2*, *SMZ*, *MAF5*, and *FLC* were involved in flowering time pathways (Fig. 5; Additional file 5).

NF-Y, a heterotrimeric TF family, consists of three subfamilies, NF-YA, NF-YB, and NF-YC. NF-YB and NF-YC form dimers with a histone-folding domain, whereas NF-YA confers sequence specificity [23, 24]. The heterotrimeric NF-Y complex binds to promoters having *CCAAT* elements and then regulates the expression of target genes [23, 24]. Although each member of the NF-Y family in yeast and mammals is encoded by a single gene, they can be spliced into multiple isoforms post-translationally modified [25, 26]. In mammals, the NF-Y complex plays important roles in many processes, including endoplasmic reticulum stress, DNA damage, and cell cycle regulation [27–29]. However, in plants, every NF-Y is encoded by multiple genes and then forms sub-families [30]. There are 10 *NF-YA*, 13 *NF-YB*, and 13 *NF-YC* genes in the *Arabidopsis* genome [31]. As with other plant TFs, duplicate members in the NF-Y family also have similar functions in *Arabidopsis* [30, 32]. The NF-Y complex plays crucial roles in plant stress responses, as well as growth and development [26, 30, 33].

NF-Y genes, including *NF-YB2*, *NF-YB3*, *NF-YC3*, *NF-YC4*, and *NF-YC9*, are involved in the photoperiod pathway of *Arabidopsis*, [34–37]. The single *nf-y* mutant did not show any obvious flowering phenotype, whereas double or triple mutants, such as *nf-yb2-1 nf-yb3-1* or *nf-yc3-2 nf-yc4-1 nf-yc9-1*, respectively, delayed flowering [37]. Because *NF-YC2* is in the same subfamily as *NF-YC3*, *NF-YC4*, and *NF-YC9*, they may possess similar functions in the photoperiod-dependent control of flowering-time. However, *tga7* exhibited a delayed-flowering phenotype under both LD and SD conditions (Fig. 1c–e), suggesting that the later flowering in *tga7* was independent of the photoperiod pathway. Thus, the decreased expression of *NF-YC2* may not result in the delayed-flowering seen in *tga7* mutant plants.

*SCHLAFMÜTZE* (*SMZ*), together with its paralog *SCHNARCHZAPFEN* (*SNZ*), belongs to the AP2-type TF family that represses flowering. Both *SMZ* and *SNZ* are targets of *miR172*, an important regulator in the ageing pathway [38]. *SMZ* delays flowering under LD conditions. When expressed in leaves, SMZ represses flowering by directly binding to the *FT* genomic locus, down-regulating *FT* expression [38, 39]. Thus, the elevated *SMZ* expression level may at least partially account for the delayed flowering of the *tga7* mutant.

Furthermore, the *FLC* and *MAF5* expression levels were increased in the *tga7* mutant compared with the WT. *FLC*, encoding an MADS-box protein, is a critical repressor in the flowering regulatory network [40–42]. MAF1–5 are five FLC homologs in *Arabidopsis*, and FLC and MAF1–5 are MADS-box TFs that repress floral transition [43]. Many flowering regulatory genes in the autonomous pathway promote flowering by directly repressing *FLC* expression, and the mutants of these genes, including *FLD* and *FLK* in the autonomous pathway, result in the delayed-flowering phenotype under both LD and SD conditions [44–46].

*FLD* encodes a histone demethylase in *Arabidopsis* and is a homolog of the human LSD1 (histone H3K4 demethylase) [47, 48]. It represses *FLC* expression through histone modifications [47–50]. *FLD* physically interacts with *FPA* and *FCA*, two autonomous pathway genes [50]. The roles of *FCA* and *FPA* on regulating *FLC* expression and floral transition may depend on *FLD* [50, 51]. Moreover, *FLD* also interacts with HDA5 and HDA6, two histone deacetylases, to regulate *FLC* expression. *FLK* contains RNA-binding domains and only exists in plants [52, 53]. FLK may repress the *FLC* expression level by binding *FLC* RNAs [54, 55]. However, how FLD and FLK regulate *FLC* expression needs further investigation. Here, we found that *TGA7* expression decreased in *fld-3* and *flk-1* mutant lines (Fig. 2d). Because the expression levels of *FLC* and *MAF5*, the closest homolog of *FLC*, increased dramatically in *tga7* compared with WT seedlings (Additional file 5, Fig. 5), and because the *tga7* mutant displayed a delayed-flowering phenotype under both LD and SD conditions (Fig. 1c, d, e), we propose that *FLD* and *FLK* regulate *FLC* expression through *TGA7*.

## Conclusions

In summary, six cDNA libraries from WT and *tga7* mutant *Arabidopsis* seedlings at 9 DAG were constructed independently for sequencing. Through bioinformatics mining, 325 DEGs were identified, and 4 genes, *NF-YC2*, *SMZ*, *MAF5*, and *FLC*, were associated with flowering time control. The differential expression levels of these flowering time-related genes were analyzed and validated by qRT-PCR. Among them, *FLC* and *MAF5* may be mainly responsible for the delayed-flowering phenotype in *tga7*, because *TGA7* expression was regulated by autonomous pathway genes. Further studies should elucidate how *TGA7* effects *FLD* and *FLK* in regulating *FLC* expression and deepen our knowledge of the autonomous pathway’s role in controlling flowering.

## Supplementary Information

**Additional file 1.** The primers used in this study.

**Additional file 2.** Alignment of WT and mutant TGA7 protein sequences.

**Additional file 3.** a The phenotypes of F_1_ seedlings grown under long-day conditions. Scale bar = 2 cm. b The cropped gels of the CAPS analysis of wild-type, *tga7 *mutant, and F_1_seedlings. Genomic DNAs of wild-type, *tga7 *mutant, and F_1_seedlings were amplified using the CAPS markers listed in Additional file 1, and then, the PCR products were digested with *Eco*RV. c The *tga7 *phenotype of F_2_ seedlings under long-day conditions. Scale bar = 2 cm. d The cropped gels of the CAPS analysis of wild-type, *tga7 *mutant, and 12 F_2_seedlings showing the *tga7* phenotype. Genomic DNAs of wild-type, *tga7 *mutant, and F_2_seedlings were amplified using the CAPS markers listed in Additional file 1, and then, the PCR products were digested with *Eco*RV.

**Additional file 4.** The detailed information of raw reads from different samples.

**Additional file 5.** Identification of key flowering time-related DEGs.

**Additional file 6.** The original, full-length gel that is displayed in Fig. 1b.

**Additional file 7.** The original, full-length gel that is displayed in Additional file 3b.

**Additional file 8.** The original, full-length gel that is displayed in Additional file 3d.

## Data Availability

The datasets supporting the conclusions of this article are available in the NCBI Short Read Archive with accession number PRJNA649868.

## Abbreviations

CAPS: Cleaved amplified polymorphic sequences
DAG: Days after germination
DEGs: Differentially expressed genes
GA: Gibberellin
GO: Gene ontology
LD: Long-day
NPR1: Non-repressor of pathogenesis-related gene 1
PCA: Principal components analysis
qRT-PCR: Quantitative real-time PCR
RNA-seq: RNA sequencing
SD: Short-day
SMZ: SCHLAFMÜTZE
SNZ: SCHNARCHZAPFEN
TF: Transcription factor
TGA: TGACG-binding transcription factor
WT: Wild-type
