# Supplementary figures and images for: Characterization of genes associated with TGA7 during the floral transition
Source: BMC Plant Biol. 2021 Aug 11;21:367. doi: 10.1186/s12870-021-03144-w (PMC8359562; doi:10.1186/s12870-021-03144-w)

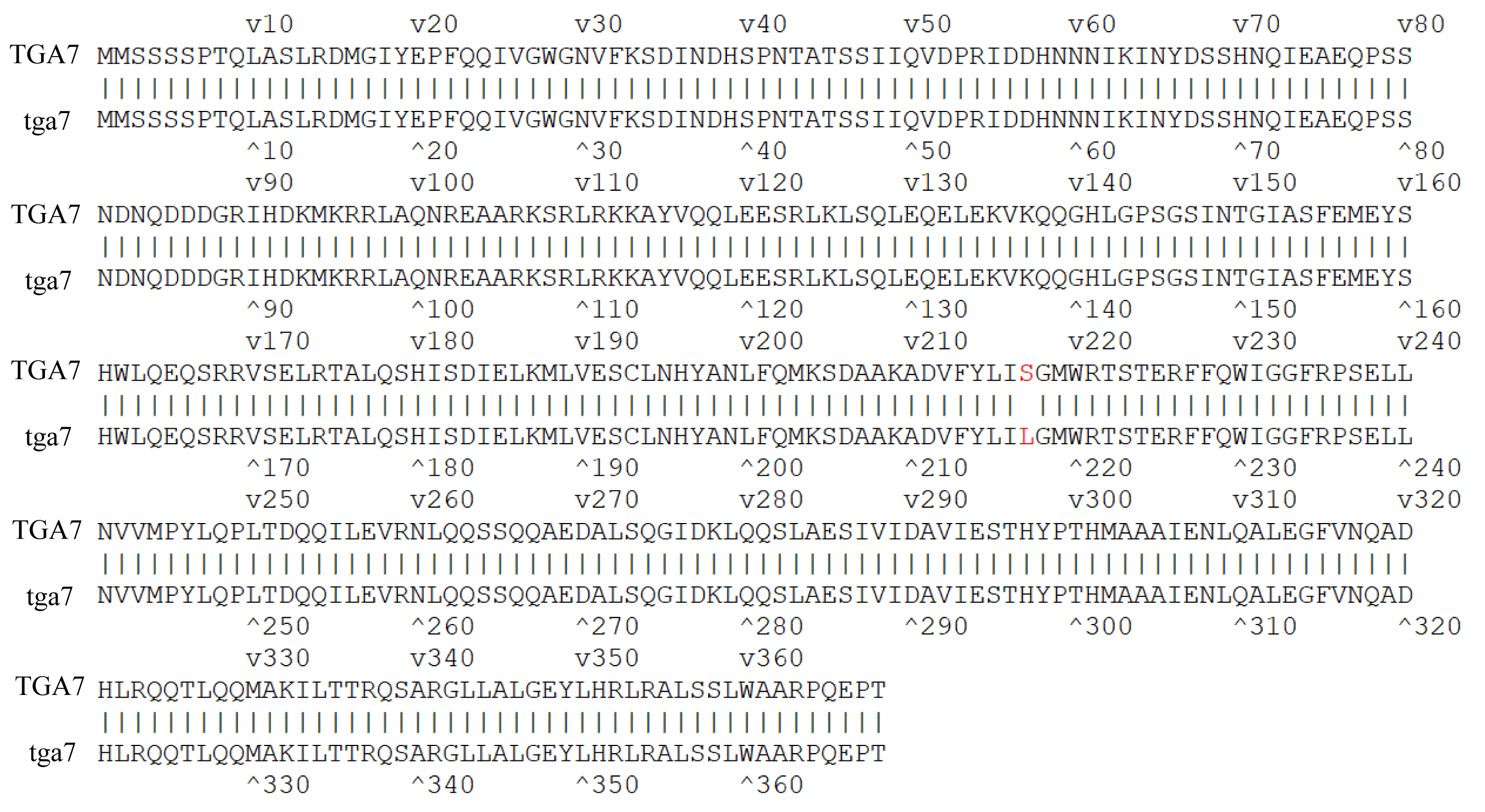

Supplement: Supplementary file 2 — Additional file 2. Alignment of WT and mutant TGA7 protein sequences. [file 12870_2021_3144_MOESM2_ESM.tif]

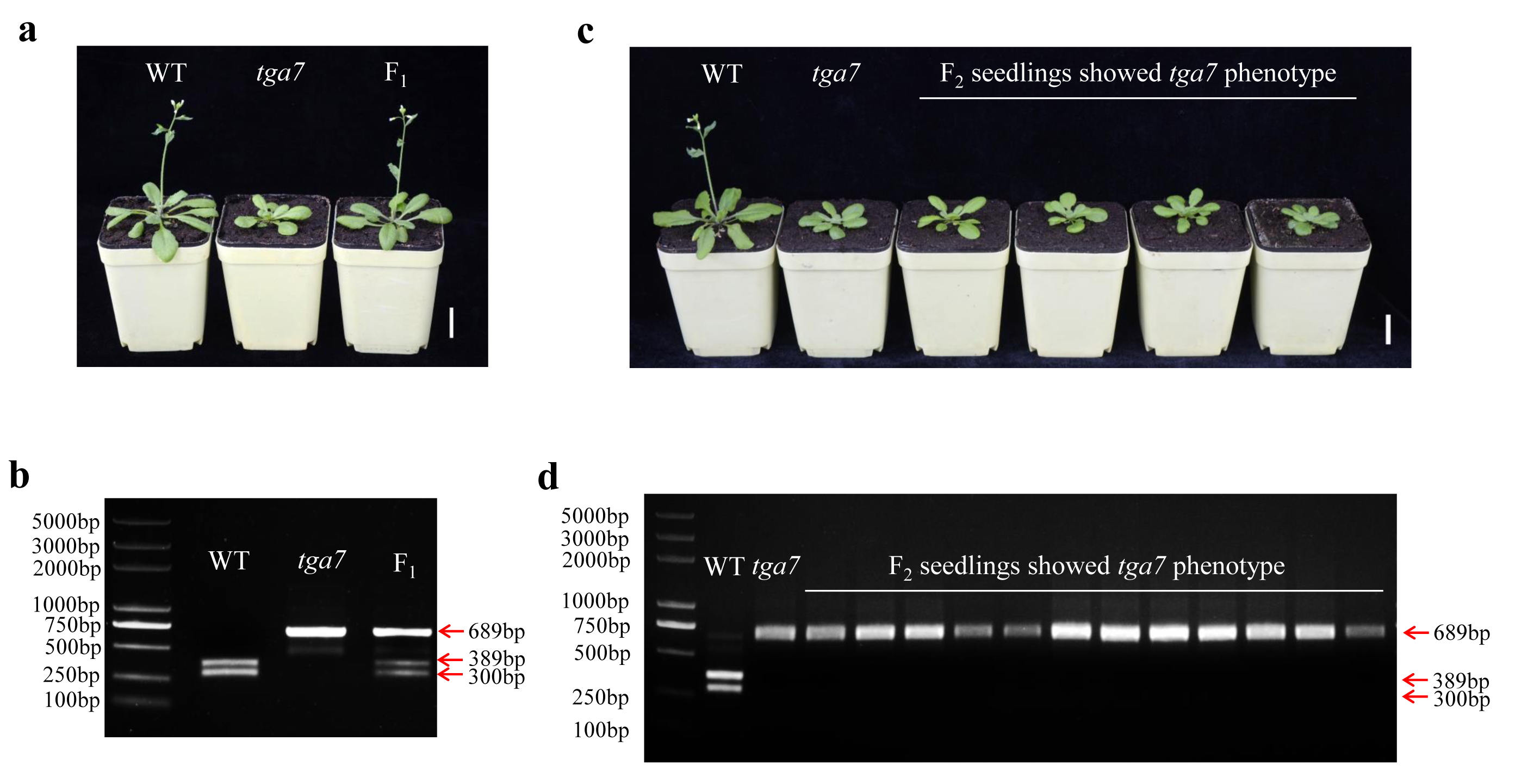

Supplement: Supplementary file 3 — Additional file 3. a The phenotypes of F1 seedlings grown under long-day conditions. Scale bar = 2 cm. b The cropped gels of the CAPS analysis of wild-type, tga7 mutant, and F1seedlings. Genomic DNAs of wild-type, tga7 mutant, and F1seedlings were amplified using the CAPS markers listed in Additional file 1, and then, the PCR products were digested with EcoRV. c The tga7 phenotype of F2 seedlings under long-day conditions. Scale bar = 2 cm. d The cropped gels of the CAPS analysis of wild-type, tga7 mutant, and 12 F2seedlings showing the tga7 phenotype. Genomic DNAs of wild-type, tga7 mutant, and F2seedlings were amplified using the CAPS markers listed in Additional file 1, and then, the PCR products were digested with EcoRV. [file 12870_2021_3144_MOESM3_ESM.tif]

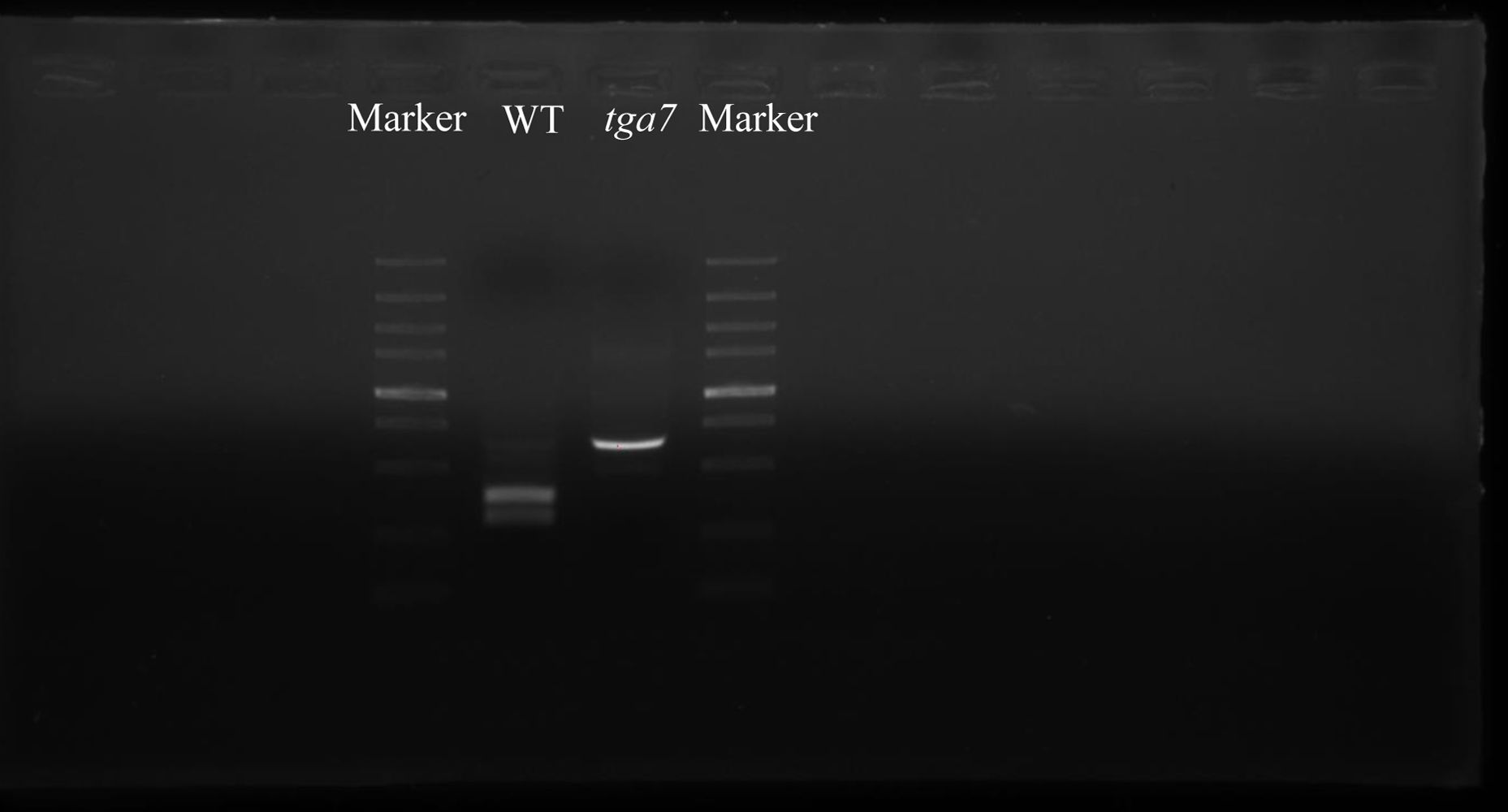

Supplement: Supplementary file 6 — Additional file 6. The original, full-length gel that is displayed in Fig. 1b. [file 12870_2021_3144_MOESM6_ESM.tif]

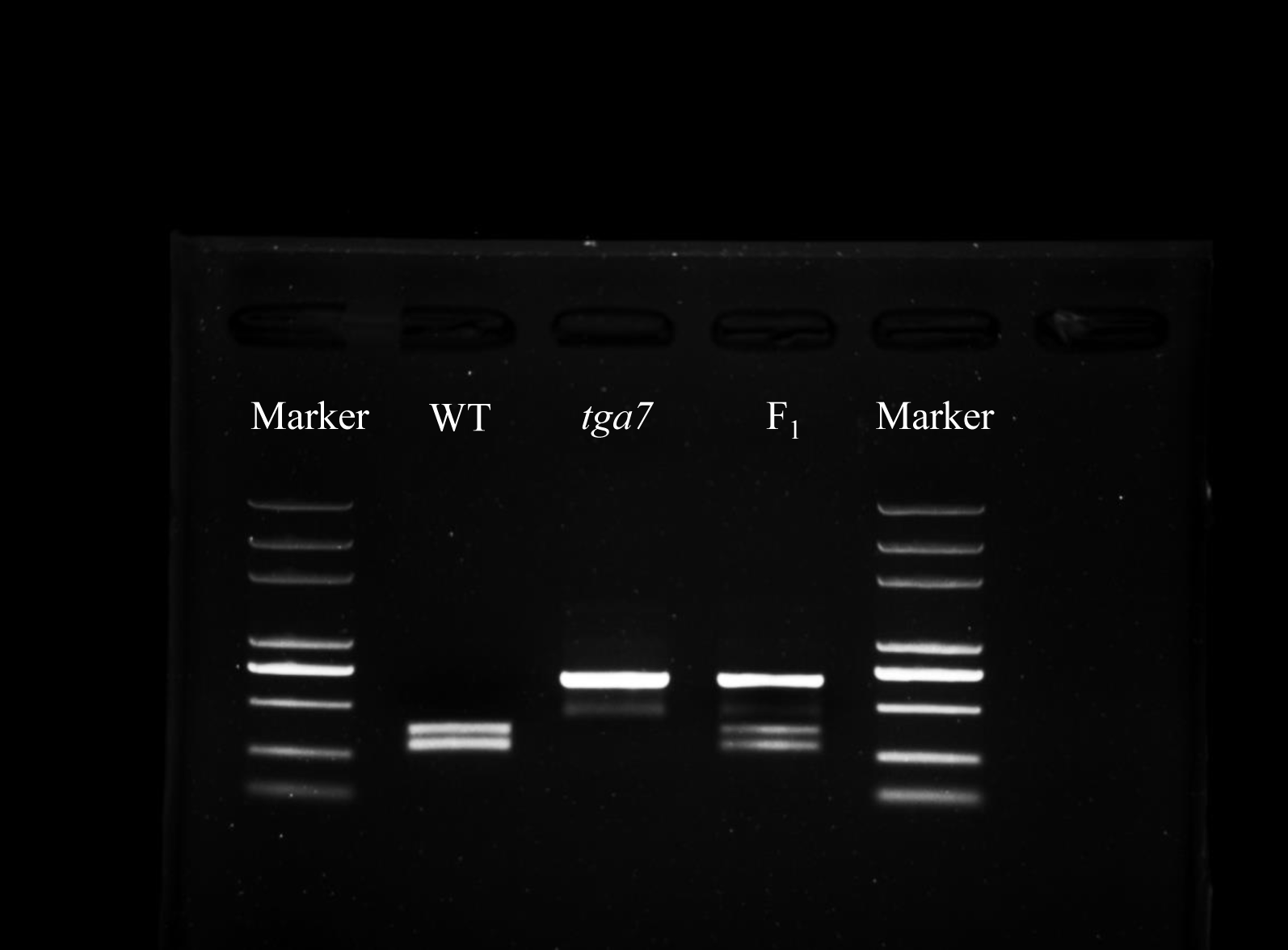

Supplement: Supplementary file 7 — Additional file 7. The original, full-length gel that is displayed in Additional file 3b. [file 12870_2021_3144_MOESM7_ESM.tif]

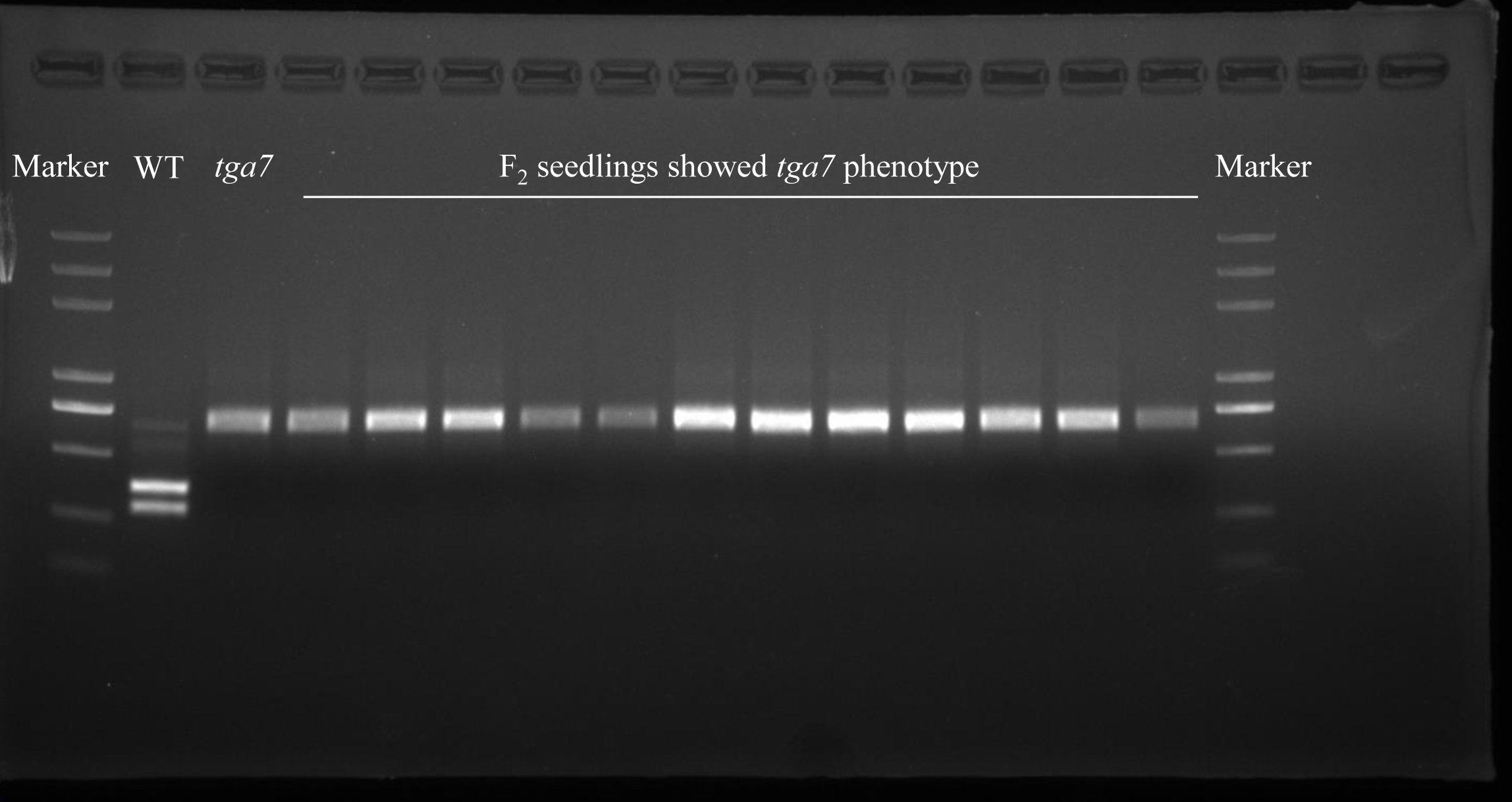

Supplement: Supplementary file 8 — Additional file 8. The original, full-length gel that is displayed in Additional file 3d. [file 12870_2021_3144_MOESM8_ESM.tif]
